# Comparative evaluation of self-etching primers with fourth and fifth generation dentin-bonding systems on carious and normal dentin substrates: An *in vitro* shear bond strength analysis

**DOI:** 10.4103/0972-0707.48839

**Published:** 2008

**Authors:** Ramesh H Giriyappa, B Suresh Chandra

**Affiliations:** Department of Conservative Dentistry and Endodontics, K. L. E. V. K. Institute of Dental Sciences, J. N. M. C. Campus, Nehrunagar, Belgaum - 590 010, Karnataka, India; Department of Conservative Dentistry and Endodontics, A. J. Institute of Dental Sciences, Kuntikana, Mangalore - 575 004, India

**Keywords:** Caries-affected dentin, fifth generation dentin-bonding system, fourth generation dentin-bonding system, self-etching primer; shear bond strength

## Abstract

**Aim:**

The aim of this study was to test the hypothesis that bonding to caries-affected dentin would yield strengths that are lower than bond strengths achievable when bonded to normal dentin. Dentin-bonding systems used in this study were fourth and fifth generation as well as self-etching primers.

**Materials and Methods:**

Forty-eight freshly extracted mandibular and maxillary molars were selected of which 24 were caries-affected teeth and the remaining were noncarious teeth. Random sampling was done with eight teeth in each group based on the bonding system used. In caries-affected teeth, the soft, stainable, caries-infected dentin was excavated using a caries detector dye whereas the hard, caries-affected, nonstainable dentin was retained. All the teeth were subsequently mounted in a suitable acrylic mould. Prepared teeth were restored with a single composite resin, using three different dentin bonding systems. These prepared specimens were transferred to a Hounsfield tensometer to measure the shear bond strength. The results obtained were analyzed using Anova, Student's unpaired t-test, and Student Neuman Keulis test.

**Results:**

The results showed that the self-etching primer required the highest mean shear load compared to the fifth and fourth generation dentin-bonding systems in both normal dentin and caries-affected dentin.

**Conclusion:**

Bond strength to dentin depends on whether the dentinal tubule is open or occluded. Within the limitations of this study, it was observed that bond strength to caries-affected dentin was low compared to normal dentin.

## INTRODUCTION

Dentin has been characterized as a biological composition of collagen matrix filled with submicron- to nanometer-sized, calcium-deficient, carbonate-rich apatite crystallites dispersed between parallel, micron-sized, hypermineralized, collagen-poor, hollow cylinders. All published reports of self-etching primers have used normal dentin as a substrate although caries-affected dentin and sclerotic, cervical dentin are more clinically relevant bonding substrates. Caries-affected dentin is the hard, sometimes stained, dentin beneath the excavated carious lesion that often forms a portion of many cavity preparations. It is not normal dentin because the tubules are often occluded with mineral crystals; however, it is free of bacteria. The mineral phase of carious dentin is often remodeled by a repetitive sequence of demineralization and remineralization, which usually produces an occlusion of tubules with mineral crystals.[1]

 The formation of the hybrid layer is believed to be due to the contact of acids with dentin, followed by adhesive resin penetration into the decalcified zone. The complete penetration of resin monomers into the demineralized dentin is essential to create strong adhesion as well as a perfect seal of enveloped collagen.[2] While there are multiple studies that examine both shear bond strength and interfacial morphology between the resin and the hybrid layer, little information is available on the bonding of the resin monomer to caries-affected dentin.

The concept of a self-etching primer was first introduced in the early 1990s in relation to dentin. Self-etching primers are expected to eliminate the risk of incomplete resin infiltration of exposed collagen fibrils scaffold with resin up to the same depth of demineralization.[3] No studies are available to date to indicate the effectiveness of self-etching primers for caries-affected dentin in comparison to normal dentin. Shear bond strength measurements are commonly used to test the effectiveness of dentin-bonding systems.

The aim of this study was to test the hypothesis that bonding to caries-affected dentin would result in lower bonding strength than when bonded to normal dentin.

## MATERIALS AND METHODS

1. Caries Detector Dye - (J. Morita)
2. Etchant - (3M)
3. All bond 2 - (Bisco)
4. Prime and Bond *NT* - (Dentsply)
5. Clearfil liner bond 2V - (Kuraray)
6. Z100 - (3M)
7. Light cure 2500 - (3M)
8. Acrylic mould
9. Teflon mould with a circular hole of 2 mm radius and 5 mm height
10. Hounsfield tensometer

### Procedure

Forty-eight freshly extracted mandibular and maxillary molars were selected of which 24 were caries-affected teeth and the other 24 were noncarious teeth. The teeth were cleared of blood, saliva, and calculus, and stored in buffered isotonic saline solution. The 24 carious teeth were randomly divided into three groups of eight teeth each, depending on the bonding system used. The inclusion criteria were: i) the caries was limited to the occlusal surface, ii) the caries extended for at least half the distance from the dentinoenamel junction to the pulp chamber, and iii) there was enough surrounding normal dentin to serve as a control bonding site. If the carious lesion was found to be shallow or deep, the tooth was excluded from the study. The occlusal surfaces of these teeth were ground to expose the carious dentin and reduced to obtain a flat surface of carious dentin that was perpendicular to the long axis of the tooth. Using a caries detector dye, all the soft, infected, stainable, carious dentin was excavated leaving behind the relatively hard, caries-affected, nonstaining dentin.

The 24 noncarious teeth were also randomly divided into eight teeth each group depending on the bonding system used. The inclusion criteria were that there should be no caries or cracks. The occlusal surfaces of these teeth were ground to expose the dentin which was flat and perpendicular to the long axis of the tooth.

**Figure 1:**
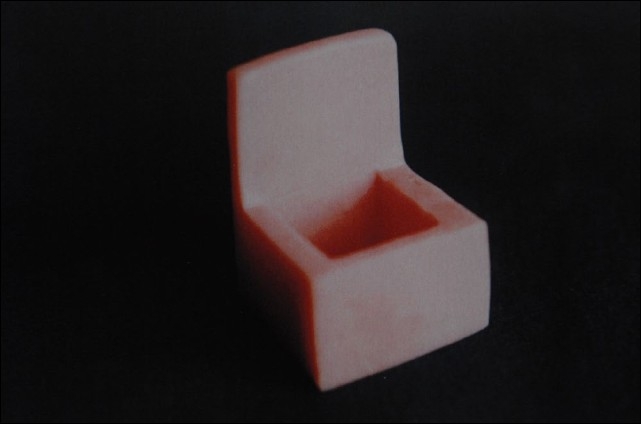
Acrylic mould

**Figure 2:**
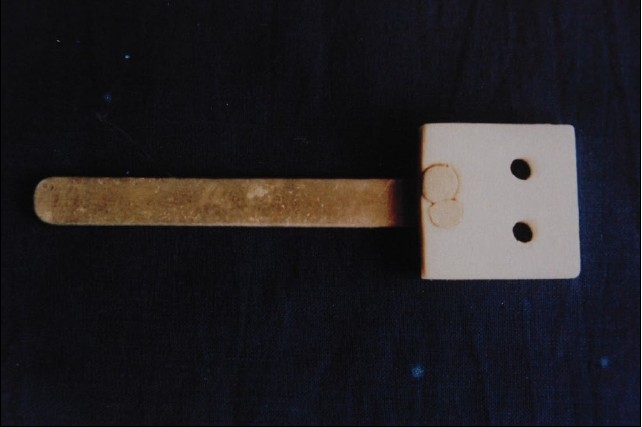
Teflon mould

Suitable acrylic moulds [Figure 1] of specific dimensions were kept ready to mount the prepared specimens. Dental stone was mixed to a fairly thick consistency and poured into the acrylic mould and the specimens thus prepared with flat surfaces were embedded in the dental stone and allowed to set. The tooth specimens were embedded such that the flat surfaces jutted out of the dental stone in the mould [Figure 2]. This procedure of preparation was repeated for all 48 specimens [Table 1].

The above procedure was carried out in specimen with normal dentin and caries affected dentin [Table 2].

**Table 1:** Operative procedure

Adhesive system	Etching	Bonding system	Restoration
All bond 2	35% phosphoric acid–15 seconds Water spray–30 seconds Gentle air drying–20 seconds	Primer applied–30 seconds. gentle blow drying 2 coats of adhesive gentle blow drying,and light–cured	All the prepared specimens were restored with single composite resin. Z100 Composite resin was inserted in increments into the circular hole of the teflon mould and light–cured. The Teflon mould was removed after curing.
Prime and bond *NT*	35% phosphoric acid–15 seconds Water spray–30 seconds Gentle air drying–20 seconds	2 coats,gentle blow drying and light–cured	
Clearfil Liner Bond 2V	Self–etching primer, apply for 30 seconds, do not wash	2 coats of bonding agent and light–cured	

**Table 2:** Shear bond strength in normal and caries-affected dentin (Mpa)

Adhesive system	Carries affected dentin	Normal dentin
All bond 2	12.25	17.5
Prime and bond NT	15.02	20.43
Clearfil liner bond 2V	16.98	22.8

### Shear bond strength study

**Figure 3:**
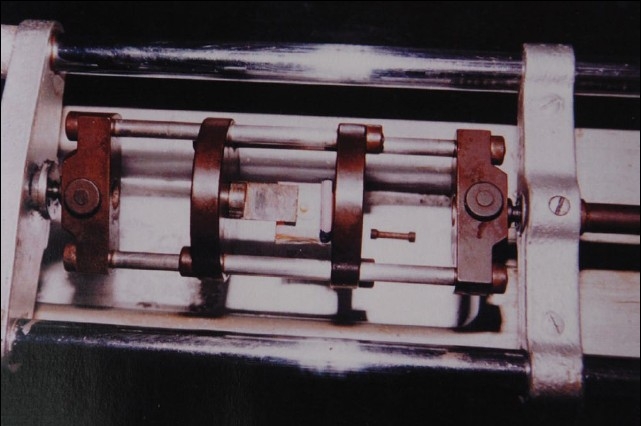
Shear bond strength study using Hounsfield tensometer

The Hounsfield tensometer is a versatile piece of equipment used to test the strength of a material. We applied the shear load with a custom-made, chisel-shaped blade by placing the acrylic moulds on the stand of the Hounsfield tensometer. The mercury level was adjusted to zero and the chisel-shaped blade was moved by fine-movement gears so as to load the sample at a steady rate. As the sample was loaded, the mercury level rose in the column, indicating the load on the sample. The cursor was moved along the level of the mercury, so that the reading indicated when the mercury level dropped at the time of sample fracture represented the shear load required to fracture the sample. All the 48 specimens were transferred to the Hounsfield tensometer individually and subjected to this shear bond strength study [Figure 3].

## RESULTS

The results were analyzed using Student's unpaired t-test, Anova (Fisher F test), Student neuman keulis test. The results revealed that Clearfil liner bond 2V required the highest mean shear load to fracture in both caries-affected dentin (16.98 MPa) and normal dentin (22.89 MPa). Prime and Bond NT required shear loads of 15.02 MPa on caries-affected dentin and 20.43 MPa on normal dentin. All bond 2 required the lowest mean shear load in both caries-affected dentin (12.25 MPa) and normal dentin (18.86 MPa). Group comparison was done using Anova and the results showed a very highly significant difference between the caries-affected dentin and the normal dentin (*P* < 0.000). Comparisons between caries-affected and normal dentin was done among the groups using the Student neuman keulis test; the results showed a very highly significant difference.

## DISCUSSION

Since the introduction of the acid etch technique into clinical practice, there has been ongoing progress in the development of more refined and diversified restorative composites along with the production of steadily improving bonding systems. Clinicians shifted from a selective etch-and-bonding technique to a total etch-and-bonding technique. The manufacturers also introduced the single-bottle resin adhesive system in an attempt to reduce the multiple steps required for bonding the composite resin to dentin. A further step towards simplifying this process saw the birth of a new generation dentin adhesive with the introduction of a self-etching primer into clinical practice, which drastically reduced the time of etching and washing.

The self-etching primer is a combination of 10-MDP (methacryloyloxydecyl dihydrogen phosphate) and 2-hydroxyethyl methacrylate along with light-activated compounds and other ingredients. Although these systems produced high bond strength with normal dentin, their bond strengths with caries affected dentin have been reported to be low. Most laboratory bonding studies are done on sound, polished, freshly cut, normal dentin. However, sound, normal dentin is frequently encountered in clinical situations; instead, clinicians are usually called for bonding to caries-affected, abraded, or sclerotic dentin. Studies on dentin permeability have revealed that after the removal of the carious lesion, the dentin was much less permeable than the intact dentin.[1]

In this study, representatives of fourth and fifth generation dentin-bonding systems were compared with a representative of the self-etching primer system group for shear bond strength studies in normal dentin and caries affected dentin. With all the three groups, we found a higher bond strength with normal dentin than with caries-affected dentin. The self-etching primer group required the highest mean shear load to fracture followed by the fifth and then the fourth generation dentin-bonding systems.

The self-etching primer created a thin hybrid layer that incorporated the smear layer. The formation of the true hybrid layer occurs irrespective of the smear layer thickness and the hybrid layer may function without separation as one unit during loading.[4] Self-etching primers superficially demineralize the normal dentin surface by partially dissolving the collagen fibrils and simultaneously permitting monomer infiltration which can be polymerized *in situ*.[5] Dentin surfaces are not washed following treatment with self-etching primer, so there is no loss of moisture from the dentin, which is thought to minimize shrinkage or collapse of the collagen fibrils network.[6] The self-etching primer adhesive system produced lower bond strengths with simulated demineralized dentin *in vitro* than with mineralized dentin covered with a smear layer.[7,8] However, these studies utilized simulated demineralized dentin rather than real caries-affected dentin. Results of the present shear bond strength study in caries-affected dentin and normal dentin is in agreement with most of the available studies, including that by Yoshiyama *et al*.[9]

Harnirattisai *et al.*,[2] studied the interfacial morphology of adhesive and etched caries-affected dentin and demonstrated the morphological variations in the resin-impregnated layer that were dependent on whether the dentinal tubules were occluded or opened. There are very few *in vitro* shear bond strength investigations that examine caries-affected dentin with newer adhesive systems, including the self-etching primer. The combination of a self-etching primer and adhesive resin into an all-in-one adhesive is advantageous in that it reduces application time and the error that may occur during each step.[10] The amorphous structure that was observed on the surface of the caries-affected dentin may affect the hybrid layer formation by forming a self-etching, primer-bonding system.[11] This could be the reason for the comparatively lower shear bond strength values with both conventional dentin-bonding and self-etching primer systems when bonded against caries-affected dentin.

CRA[12] reported shear bond strengths up to 21.1 MPa for Clearfil liner bond 2V which is in agreement with our results with normal dentin. However, clinical reports with caries-affected dentin are not available for Clearfil liner bond 2V. Two of the three self-etching primers evaluated had lower bond strength to caries-affected dentin than to normal dentin,[8] which is in agreement with our results. Nakajima suggested that when one is dealing clinically with complex cavity preparation, technique sensitivity of the adhesive system might be a major factor in limiting longevity of the restoration.

The results of our study clearly indicated that the self-etching primer tested within the parameters of this study demonstrated superior bond strength both with normal and caries-affected dentin. The conventional bonding systems tested in this study also demonstrated fairly comparable, clinically acceptable values against both normal and caries-affected dentin. There is a need especially for *in vivo* studies, preferably with human subjects, to evaluate the long-term durability of improved materials to clinically relevant substrates, including normal and sclerotic dentin. Therefore, adhesive resin systems that require simple procedures and high bond strength to any substrate independent of depth, region, and mineralization of substances remain a necessity for an ultimate adhesive system. Further studies of clinically relevant substrates and preparation configuration are necessary.

## CONCLUSION

The present *in vitro* investigation evaluated the shear bond strength of caries-affected and normal dentin restored with a single composite resin (Z100) in conjunction with Group I (All bond 2), Group II (Prime and bond NT), and Group III (Clearfil liner bond 2V). The following conclusions were drawn:

1. In the present study, Group III fractured under the highest mean shear load, whereas Group I recorded the lowest shear load in both caries-affected and normal dentin.
2. Group III recorded higher mean shear load values than Group II, followed by Group I.
3. Bond strength to caries-affected dentin was substantially lower compared to normal dentin in all the three dentin-bonding systems that were compared.

## References

[1] Pashley DH (1991). Permeability of normal vs carious dentine. Endo Dent Traumatol.

[2] Harnirattisai C (1992). Interfacial morphology of an adhesive composite resin and etched caries affected dentin. Oper Dent.

[3] Prati C, Chersoni S, Mongiorgi R, Pashley DH (1998). Resin infiltrated dentin layer formation of new bonding system. Oper Dent.

[4] Pashley DH, Sano H (2000). Effects of smear layers on the bonding of self etching primer to dentin. J Adhes Dent.

[5] Van Meerbeek B, Perdiago J (1998). The clinical performances of adhesives. J Dent.

[6] Nakajima M, Sano H (2000). Bond strength of single bottle dentin adhesive to caries affected dentin. Oper Dent.

[7] Nakajima M, Ogata M, Harada N, Tagami J, Pashley DH (2000). Bond strengths of self etching primer adhesives to in-vitro demineralised dentin following mineralizing treatment. J Adhes Dent.

[8] Nakajima M, Ogata M, Okuda M, Tagami J, Sano H, Pashley DH (1999). Bonding to caries affected dentin using self etching primers. Am J Dent.

[9] Yoshiyama M, Urayama A, Kimochi T, Matsuo T, Pashley DH (2000). Comparison of conventional vs self etching adhesive bonds to caries affected dentin. Oper Dent.

[10] Tay FR, Pashley DH (2001). Aggressiveness of contemporary self etching systems I: Depth of penetration beyond dentin smear layers. Dent Mater.

[11] Yoshiyama M, Urayama A, Kimochi T, Matsuo T, Pashley DH (2000). Comparison of conventional vs self etching adhesive bonds to caries affected dentin. Oper Dent.

[12] CRA Report.

[13] Ogata M, Harada N, Yamaguchi S, Nakajima M, Pereira PN, Tagami J (2001). Effect of different burs on dentin bond strength of self etching primer bonding system. Oper Dent.

[14] Tagami J, Tao L, Pashley DH, Hosoda H, Sano H (1991). Effects of high speed cutting on dentin permeability. Dent Mater.

